# GECKO: a complete large-scale gene expression analysis platform

**DOI:** 10.1186/1471-2105-5-195

**Published:** 2004-12-10

**Authors:** Joachim Theilhaber, Anatoly Ulyanov, Anish Malanthara, Jack Cole, Dapeng Xu, Robert Nahf, Michael Heuer, Christoph Brockel, Steven Bushnell

**Affiliations:** 1 Cambridge Genomics Center, Sanofi-Aventis, 26 Landsdowne Street, Cambridge, MA 02139, USA; 2 Sanofi-Aventis, Genomics and Scientific Computation, Route 202–206, Bridgewater, NJ 08807, USA; 3 Fast Gun Software, Inc., 180 Myrtle St., Wrentham MA, 02093, USA; 4 Sanofi-Aventis Tucson Selectide, 1580 E. Hanley Blvd., Tucson, AZ 85737, USA; 5 Center for Computational Genomics and Bioinformatics, University of Minnesota, 426 Church Street SE, Minneapolis, MN 55455, USA

## Abstract

**Background:**

Gecko (Gene Expression: Computation and Knowledge Organization) is a complete, high-capacity centralized gene expression analysis system, developed in response to the needs of a distributed user community.

**Results:**

Based on a client-server architecture, with a centralized repository of typically many tens of thousands of Affymetrix scans, Gecko includes automatic processing pipelines for uploading data from remote sites, a data base, a computational engine implementing ~ 50 different analysis tools, and a client application. Among available analysis tools are clustering methods, principal component analysis, supervised classification including feature selection and cross-validation, multi-factorial ANOVA, statistical contrast calculations, and various post-processing tools for extracting data at given error rates or significance levels. On account of its open architecture, Gecko also allows for the integration of new algorithms. The Gecko framework is very general: non-Affymetrix and non-gene expression data can be analyzed as well. A unique feature of the Gecko architecture is the concept of the Analysis Tree (actually, a directed acyclic graph), in which all successive results in ongoing analyses are saved. This approach has proven invaluable in allowing a large (~ 100 users) and distributed community to share results, and to repeatedly return over a span of years to older and potentially very complex analyses of gene expression data.

**Conclusions:**

The Gecko system is being made publicly available as free software . In totality or in parts, the Gecko framework should prove useful to users and system developers with a broad range of analysis needs.

## Background

**Figure 1:**
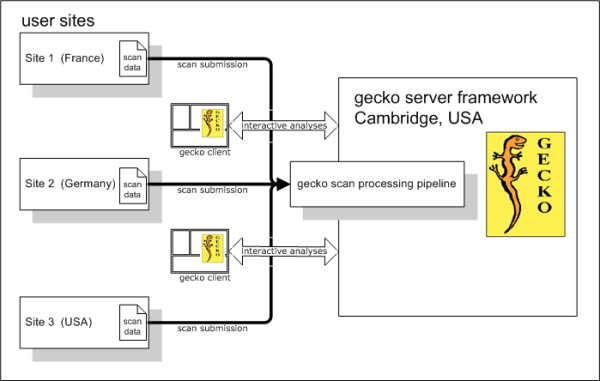
**Sketch of the functional organization of a Gecko installation**, emphasizing its distributed aspects. Research groups in France, Germany and the United States (sites #1 through #3) submit Affymetrix scan data (3 dark lines) to a server based in Cambridge, United States. Users can conduct analyses on any part of the stored data, using a client application (small Gecko icons) which enables two-way communication with the server (two-way arrows).

In recent years, in response to the needs of our scientific community we have developed a comprehensive, company-wide gene expression data analysis platform based on a centralized client-server architecture (Figure 1). This platform, named Gecko (Gene Expression: Computation and Knowledge Organization) addresses the problems of analyzing large volumes of continuously generated data (thousands of Affymetrix scans per year), provides a broad spectrum of analysis tools, and creates a single, collaborative view of data for a large, decentralized community of users.

Three organizing concepts have guided the construction of Gecko. The first is the use of the *Analysis Tree *(actually, a directed acyclic graph) which provides a complete historical and hierarchical display of all analyses conducted to date by the users. In particular, in support of this concept, Gecko permanently stores the results of *all *analyses performed. A second organizing concept is that of the *agglomeration *syntax, an "Erector Set" of operations for flexibly creating, combining and subsetting data matrices. The third organizing concept is the pervasive use of *experimental designs*, which are associated with each data matrix, and which enable the application of a wide range of statistical and pattern recognition tools.

It is the aim of this paper to give an idea of the user's view of Gecko and how one conducts analyses using the system, as well as to provide a software-level overview of the Gecko system architecture. Indeed, we believe that Gecko presents a number of innovative features well-worth presenting, and in connection with this publication, we are making available a public release of the Gecko software[1].

In what follows, we first go "behind the scenes", and present the system architecture in some detail, including overall data organization, database structure, computational engines, statistical tools and models, and finally utility programs. We then present a focused discussion of a specific analysis example, so as to give the reader a more immediate impression of the Gecko system.

## Implementation

### The Gecko architecture

**Figure 2:**
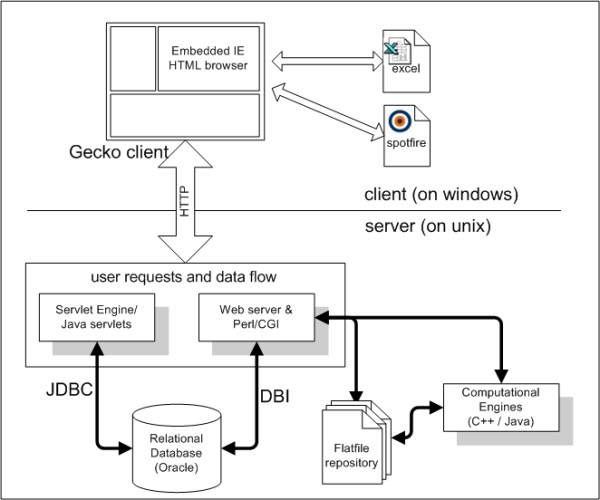
**Overview of the Gecko software architecture**, including the major components of the client and server. Note that the client allows for data streaming to other applications, such as Excel or Spotfire.

Gecko is based on a client-server architecture, with a global structure shown in Figure 2. The Gecko users have remote access to the system through a client application, currently designed for the Windows operating system and running on any desktop or laptop computer (a prototype Java-based client has also been developed, but is not yet in production use). Overall, the Gecko client is a "thin" client, focused on handling user requests and server responses, with most of the actual computation and data organizational tasks handled by the Gecko server. As indicated in the figure, the client not only manages interaction with the Gecko server, but also allows for local connection to applications such as Microsoft Excel or the Spotfire visualization tools[2], which can be invoked for additional data analysis after the data has been automatically streamed to these applications from the client.

The exchanges between the client and the server occur through HTTP requests, which transit through a web server running on the server platform. A central aspect of the Gecko client is that it contains an embedded Internet Explorer browser, a feature which greatly simplifies the task of building user interfaces. Thus, forms for submitting parameters to the server are typically built in HTML dynamically generated by server-side Perl CGI or Java servlet programs, and displayed in the embedded browser.

The Gecko server itself runs on a UNIX platform, and consists of the four main components indicated in Figure 2: a database, that predominantly contains non-numerical, organizational data; a set of computational engines, written in C++, Java, or Perl; a set of request-handler programs (Perl CGI and Java servlet programs) that enable the client-server interaction; and a flat file repository, that contain files for both raw numerical expression data (scans) as well as for all derived data types (analyses).

### The Gecko database

The set of tables in the Gecko database can be partitioned into three main groups, which we call the "Scan", "Chip" and "Analysis" groups in accordance to their functional roles.

The Scan group of tables stores attributes of the individual scans of microarray data entered into the system. These attributes include a unique scan identifier (the scan name), as well as many parameters (project name, experiment name, sample name, compound(s) applied and treatment duration, hybridization protocols, etc), which record the nature of the biological sample used and how it was processed, and place the scan in a tree with experimental and biological context.

While the Scan group of tables captures many items in common with the so-called MIAME (Minimum Information about a Microarray Experiment) annotation standards[3], it should be emphasized that its design antecedates the creation of the MIAME standards, and is neither as comprehensive, nor fully consistent with these standards. In current installations of Gecko, an independent laboratory information management system (LIMS), upstream of the Gecko analysis platform itself, provides considerably more detailed information about the samples. With our emphasis on Gecko as an analysis platform and not as a LIMS, we have so far deferred the question of how to best federate (under a MIAME-compliant heading) all of the experimental annotation information.

The Scan group of tables also records numerical data in the form of summary statistics for each scan, including several measures of chip brightness and measures of noise and saturation. However, the bulk numerical data for each scan is stored as a file in the flat file repository, with only a file pointer stored in the database.

The Chip group of tables stores the attributes of the Affymetrix chip designs currently known to the system. These include the names of the chip designs used, and for each chip design, all the qualifiers pertaining to it. The tables also store sequence annotation information on a qualifier-by-qualifier basis, including a short description line, as well as a URL that provides a link to more general annotation information for each qualifier. The annotation information is generated externally to the Gecko system, with periodic updates via flat files which can be automatically uploaded.

The Analysis group of tables is central to all the analysis functions available in Gecko. Any analysis object generated in the system has a set of attributes which are saved under five categories of information: 1) its parent/child relations, 2) an internal pointer to the machine-generated file which contains the bulk numerical data, 3) the parameters for the operation which created the analysis object, 4) general parameters (name of the analysis object, data type, number of rows and columns in the data matrix, etc), and 5) experimental design parameters. Knowledge of the experimental design [[4], p.93] [[5], p.214] underlying a dataset is essential to many types of analyses (e.g. ANOVA, contrast calculations [[5], p.214], supervised classification). In Gecko, the experimental design parameters (factors and levels) of each data matrix are thus stored in the database, and can be accessed or modified by the user at any time. They are automatically retrieved and used whenever a relevant analysis is invoked.

Finally we note that no access control is imposed in Gecko: any user can access any collection of scans, or visit any of the existing analyses created by other users. While this very open architecture has greatly fostered collaboration, it is conceivable that access control might eventually be required. To that end, limited architectural and programming modifications are needed. Modifications might consist of expansion of the current user tables, to include group definition and password fields, and addition of straightforward programming logic in both client and server, to mask access to data which is out of the scope of a given user.

### Computational engines

Gecko incorporates a spectrum of computational tools, which enter into 5 major categories: 1) agglomeration, 2) statistical analysis, 3) clustering, 4) supervised classification and 5) transformation methods

#### Agglomeration tools

**Figure 3:**
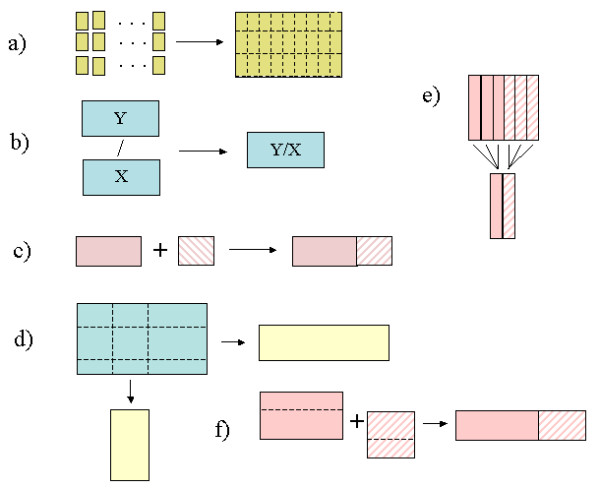
**Examples of the Gecko ***agglomeration syntax*. a) *Concatenate*: concatenation of a large number of scans (here arising from many samples, each profiled across three distinct chip designs) into a single data matrix; normalizations are computed on-the-fly; b) *Cat Ratio*: element-by-element ratios are computed for two data matrices, creating a new data matrix containing ratios and P-values; c) *Combine on Columns*: the columns of two data matrices are concatenated to create a larger data matrix; d) *Reduce on Qlist *and *Reduce on Columns*: a data matrix is subsetted on its rows (right-hand arrow) or on its columns (bottom arrow), respectively, to form reduced data matrices; e) *Merge Replicates*: replicates are merged by taking medians of intensities (with concomitant reestimation of noise terms); f) *Join*: two data matrices are joined using qualifiers (row indices) as the join key.

Data for a given experiment is typically distributed over many scans, numbering in some cases hundreds or even thousands. The ability to easily construct or modify the relevant data matrix, with appropriate normalization of scans with respect to each other, is thus critical to all downstream analysis, and this need is addressed by a suite of tools under the generic heading of *agglomeration *(Figure 3). For instance, the Gecko *Concatenate *tool enables assembly of large sets of scans (Figure 3a) through the submission of a simple spreadsheet containing the list of scans in an ordered format. The spreadsheet data entry optionally includes specification of the experimental design (factors and levels), which can also be modified or created de novo at any later time.

Once created, a data matrix then becomes accessible as a single object, to be used in higher-level agglomeration operations. For instance, *Cat Ratio *enables one to take ratios of two complete data matrices, on a element-by-element basis (Figure 3b). To achieve this, the user needs only to specify the two relevant datasets by selecting the corresponding nodes in the Analysis Tree. All subsequent aspects of the computation (matching numerators and denominators pairwise, actual ratio calculations, on-the-fly normalizations, etc) are achieved automatically, and the resulting data matrix, now containing ratios, is registered in Gecko as a child of the two input datasets.

The suite of agglomeration operations also includes concatenating data matrices to each other, merging replicates within an agglomerated dataset, subsetting on rows or columns, or performing join operations (Figs. 3c,3d,3e), altogether approximating an "Erector Set" for building data matrices out of smaller or larger blocks. These operations are routinely performed on large datasets (currently up to ~ 50000 rows × 500 columns). To indicate processing times for these operations, we note that on a 400 MHz Sun Enterprise server, concatenation requires about 2 second per scan, while the more complex ratio calculations require about 10 seconds per scan pair. Thus concatenating, say, 1000 scans, will require about 30 minutes of processing time, while computing the ratio of 1000 scans to another 1000 scans simultaneously, will require about 3 hours of processing time.

We finally note that users can bypass agglomeration of scan data altogether, and directly upload arbitrary data matrices into the system (see *Data sources *section below). This feature makes Gecko into a general analysis tool, for multivariate analysis in contexts quite different from that of gene expression.

#### Statistical analysis tools

The suite of statistical analysis tools includes application of both parametric and non-parametric tests to the agglomerated data matrices, on a qualifier-by-qualifier basis and using the associated experimental designs. Included are two-class comparison tests (Student t-tests, SAM[6], comparison of variances, Mann-Whitney [[5], p.265]), as well as multiple-class and multiple-factors tests (one and two-way ANOVA) and the ability to perform contrast calculations [[5], p. 241] of several different types.

The parametric tests are available with a "renormalization" option which corrects P-values in accordance to an intra-class correlation (icc) model (JT, manuscript to be submitted for publication). For instance, when applied to a one-way ANOVA across several classes, the icc model folds part of the class-dependent effects into the null hypothesis, by mathematically assuming that they have a random component already explained by the null hypothesis, with variance proportional to the variance of the residuals within each class. The proportionality constant is then computed on-the-fly, by requiring that the resulting distribution of the *F *statistic over all genes is non-significant up to its median value. This renormalization suppresses weak or biologically unremarkable class-dependent effects, while preserving significant data in the upper tail of the observed *F *distribution. It typically avoids the conundrum of "all genes are significantly regulated" which very often occurs as the number of samples becomes large.

Biased-variance versions of the parametric tests (where an additional, fixed variance term is introduced in the denominators of the *t *or *F *statistics so as to reduce noise) are also implemented, in a form where the icc model is combined with semi-parametric resampling to estimate accurate P-values.

Alongside these statistical location tests, which depend on samples being assigned to different classes, one can compute class-independent statistics, such as χ^2^, grand means or standard deviations on a qualifier-by-qualifier basis across all samples. These tests are frequently useful in ranking expression profiles on the basis of one or several of these test statistics, typically for subsequent filtering-out of noisy profiles, or for overall statistical assessment of the dataset.

Tests incorporating the calculation of the Pearson correlation coefficient are also implemented. These tests enable one to perform "nearest-neighbor" searches for the expression profiles most like those of single or multiple query profiles. As with the set of location tests, these correlation-based tests include options for renormalization, based on the icc model, and for biased-variance terms in the denominators of the equations for correlation coefficients.

As an indication of typical execution times for statistical tests, we note that on a 400 MHz Sun Enterprise server, a two-way ANOVA with associated contrasts, applied to a ~ 22000 rows × 100 columns data matrix, requires about 120 seconds of processing time.

In all cases, tests results are saved in the Gecko Analysis Tree and can be revisited a posteriori by use of the generic *Get Stats *tool, which internally computes receiver operating characteristics (ROCs) [[7], p. 48], generates graphics for the corresponding ROC plots, and allows for selection of qualifiers based on P-value or on false-discovery rate criteria[8].

#### Clustering and supervised classification tools

The types of clustering tools implemented in Gecko include self-organized maps (SOM)[9], average linkage hierarchical clustering [[10], p. 318], principal component analysis (PCA) [[11], p. 23], multidimensional scaling (MDS) [[11], p. 107] and the ability to build and display correlation or distance matrices. Supervised classification tools include a gene expression *k*-nearest-neighbor classifier(GENNC)[12], in conjunction with fully self-consistent feature selection, based on a number of cross-validation methods (leave-one-out, leave-one-group-out, v-fold) [[13], p. 219].

#### Transformations

Data transformations are frequently required in the course of analyses. Among those available in Gecko are point transformations, where each element of the data matrix is independently transformed (log-transformations, flooring of values to the noise standard deviation, and others), as well as more global transformations, including variance stabilization[14], standardization of rows and/or columns (by mean or median centering followed by division by the corresponding standard deviations) [[11], p. 8], and wholesale transposition of the data matrix. In Gecko, transformations usually appear as explicit steps in the Analysis Tree, rather than being "rolled into" other operations, such as clustering.

#### Adding new analysis methods

New analysis methods, if already available as executables or applications running from the UNIX command line (for instance, based on C++, Java, R, Matlab, or other languages), can be internally added to the Gecko system by straightforward programming steps. These steps include i) providing for a user interface, generated by server Perl CGI or Java servlet programs, and displayed as HTML in the client Browser window; and ii) constructing a server-based driver program, that will execute the UNIX command, using the parameters communicated by the user interface. We note that while an application programming interface (API) has not been formalized, a Gecko API is already well-approximated, by the existence of a modular set of methods for accessing the database, and for reading and writing to numerical flat files.

For external analysis using other applications, direct streaming of all internal Gecko types is currently implemented for Spotfire[2] and Microsoft Excel. For saving data to local disk, generic data export in tab-separated values format is also possible. Furthermore, specially formatted types of data export to disk have also been implemented, in particular for the Cluster and TreeView[15] clustering and visualization programs. Extending the number of specially formatted export options to other analysis packages (for instance, to create R "data frames" to be used in BioConductor R packages[16]), should be a straightforward programming task, consisting of adding an appropriate formatting function to the existing Perl/CGI module.

### Data organization in Gecko: the Analysis Tree

A central concern in the design of Gecko was to enable the user to perform and especially to later recall complex analysis work flows (such as the cell line data analysis, described in detail below). In general, graphs of analyses conducted in Gecko, with nodes corresponding to datasets and edges to operations on these datasets, result in directed acyclic graphs (DAGs). A DAG is unlike a tree, in that each of its nodes can have multiple parents, whereas in a tree each node has a unique parent; for simplicity however, we refer to the DAG generated by Gecko as the Analysis "Tree". Furthermore, in the Gecko client the DAG is actually displayed as a tree: the DAG topology is correctly maintained by replicating, for nodes with multiple parents, the corresponding subgraphs under each of the parent nodes.

**Figure 4:**
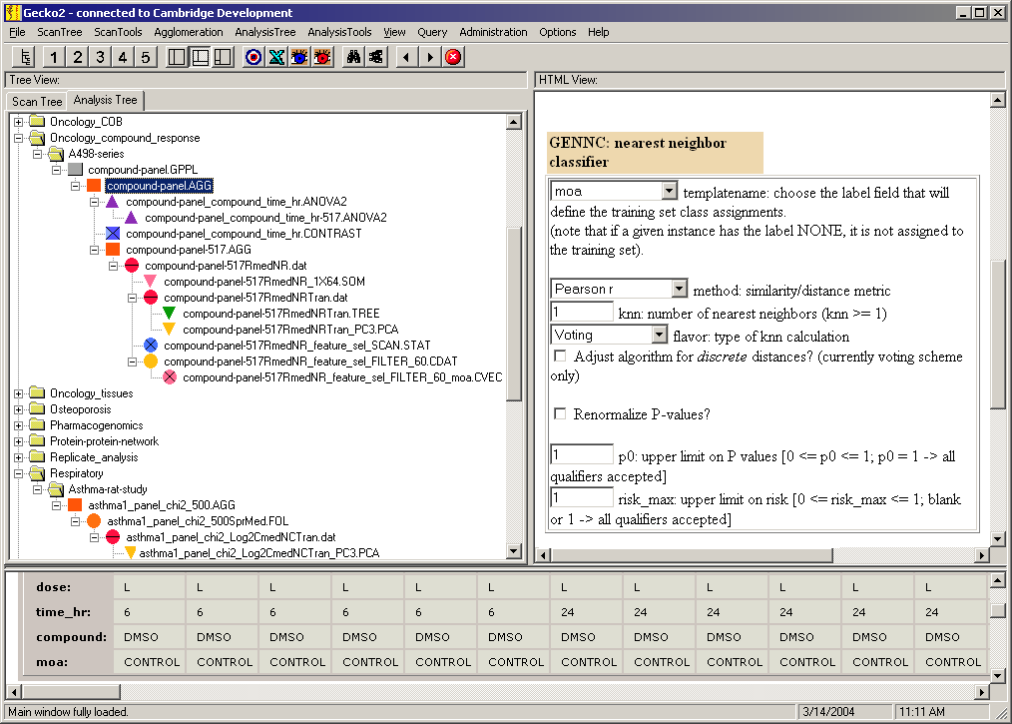
**The Gecko user interface**, showing Analysis Tree (upper left-hand-side), Browser (upper right-hand-side) and Properties windows (bottom). The Analysis Tree is opened on the A498 cell line data analyses described in the text (top), as well as on another independent study (bottom). An input form for a *k*-nearest-neighbor classifier is displayed in the Browser window. The experimental design for **compound_panel.AGG **(the selected object) is displayed in the Properties window.

Once generated, the data file corresponding to a node in the Gecko Analysis Tree is permanently stored (unless the node is explicitly deleted by the user at some later time). This approach enables users to return at any time to potentially very large and complex panels of analysis results, without requiring them to regenerate all final and intermediate results on-the-fly, as might be required in an alternative real-time "dataflow" approach (in which only the sequence of operations is permanently stored, and in which data is recomputed every time a new session is started). We have found that the dataflow approach can entail a prohibitive computational cost and waiting time, whenever a large number of analyses are being simultaneously considered, as in the examples of Figure 4 (described in detail below). This situation is obviously exacerbated by the presence of individual lengthy computations, such as are required for instance for classifier cross-validation.

The permanent storage of all analysis results might seem an extravagant use of computer resources, but experience shows that it results in reasonable use of server memory over time. For an expression analysis community of roughly 100 scientific users, over a span of 5 years memory use has been limited to about 150 GB (corresponding to the disk space available on a couple of current generation personal computers), reached with slow linear growth over time. Furthermore, should it be absolutely required, implementing a file archival and retrieval system for the oldest analyses would be a straightforward task.

### Noise model

The Gecko noise model is based on the so-called PFOLD joint noise model and ratio estimation algorithm[17]. This model includes both additive (background, cross-hybridization) and multiplicative (coefficient-of-variation effects) noise terms, within a Bayesian estimation framework. Expression ratios and related P-values and confidence limits are computed on the basis of a posterior distribution of ratios conditional on measured intensities and noise terms. The rigorous mathematical derivation of the posterior distribution results in a formulation that seamlessly connects high and low signal-to-noise regimes, and allows estimation of ratios even when recorded intensities are zero or negative.

### Data sources

While currently all scans uploaded into Gecko are generated by Affymetrix technology, in the past the system has also been used with other types of expression data, for instance generated by two-color hybridizations on spotted arrays. This has been possible at low programming cost, because the internal representation of scan data in Gecko is *independent *of microarray technology, with a generalized storage of intensity and noise information for each chip qualifier or microarray spot. Programming modifications needed for a new technology thus primarily occur in the design of the new raw-file parser (automatically invoked on entry by the scan processing pipeline). Note that for the two-color technologies mentioned above, data for each channel is entered as a separate intensity scan. Channel-to-channel ratios between matched scans are then computed downstream by the users, using the *Cat Ratio *Agglomeration tool mentioned above.

An alternative and very flexible method for data entry into Gecko, which entirely bypasses scan entry, is to directly upload a tabular file through the Gecko client. In particular, this method enables one to upload gene expression data from the many public sources where it is provided only in spreadsheet format. Furthermore, as already stated, it also enables one to use the Gecko analysis tools in contexts unrelated to gene expression.

### Utilities: the Gecko scan processing pipeline

As Gecko was designed as a centralized resource, but also for service of geographically remote sites (Figure 1), it was critical that the Affymetrix scan submission process be made as automatic and foolproof as possible. To that end, a two-step procedure was devised, described as follows.

First, users register scans through an interface provided in the Gecko client, using an appropriate submission window. This registration step stores the scan attributes in the Gecko database (project name, experiment name, sample name, and so forth), but does not transfer the scan numerical data (intensity values) itself. In the second, independent step, the users send the scan numerical data, in the form of Affymetrix CEL files[18], to a specific incoming directory on the Gecko server, typically using the file transfer protocol (FTP) utility (Figure 1).

The Gecko scan processing pipeline, run as a periodic "cron" job on the UNIX platform, automatically converts the Affymetrix CEL file data to Affymetrix MAS5[18] estimated values, using an emulator of the corresponding algorithm, and writes the results in a format specific to the Gecko system, finally setting a "processing pending" flag to off for each processed scan. Error statuses for files which exceptionally fail processing are written into the database and displayed in a client-based processing queue administration window. On a 400 MHz Sun Enterprise server, the processing time per scan is approximately 3 minutes, enabling upload of about 500 scans per 24 hour period.

The processing pipeline has proven to be very robust, and can be readily modified to accept other sources of gene expression data, as already mentioned above. Thus, it has also been used to process cDNA microarray data[19] in the past.

## Results

### An analysis example

As an example of an analysis workflow conducted in Gecko, we describe a study of a cancer cell line treated with a panel of compounds which are inhibitors of cell proliferation. Cultures of the A498 cell line (a cell line derived from kidney carcinoma and part of the NCI60 panel[20]) were treated with five different dimethyl sulfoxide(DMSO)-dissolved compounds (here named A1, A2, A3, B1 and B2) falling into two distinct classes (DNA replication inhibition or tubulin binding, A and B, respectively) depending on their mechanism of action. Control cell cultures, treated with the DMSO solvent alone, were also generated. Six biological replicates of the cell cultures were generated for each combination of compound and harvest time, with harvests occurring at 6 hours or 24 hours after the start of treatment. After processing of the cell extracts, the resulting cRNA samples were hybridized to HG_U133A Affymetrix chips[18,21], resulting in a total of 72 chip scans, which were submitted to the Gecko scan processing pipeline, and uploaded into the system.

### The Gecko client user interface

Figure 4 shows the Gecko client as seen by the user. The client user interface consists of a list of menu items (top), with an associated list of icons (shortcuts to menu items, immediately below), under which are three large adjustable window panes, with content as follows.

The left-hand window pane (Tree window) provides a tree representation of the data objects existing in Gecko; in the figure, it currently displays the Analysis Tree, which provides a full and permanent record of analysis operations and resulting datasets executed so far. This window can also display the Scan Tree, a hierarchical display of all scans in the system, by selection of the corresponding Scan Tree tab (upper left-hand corner).

The right-hand window pane of the client (the Browser window) contains forms for submitting parameters to the analysis tools, and also displays analysis results. Currently selected is an input form for performing supervised classification of the compound-treated samples, using a *k*-nearest neighbor classifier[12].

The bottom window pane of the client (the Properties window) displays the properties of the object currently selected in the Analysis Tree. Here, the experimental design for the selected object, the data matrix **compound-panel.AGG**, is currently visible.

The Analysis Tree contains nodes at three types of levels. Nodes at the highest, most general level are named Projects: in Figure 4, the Analysis Tree is opened under the Project **Oncology_compound_response**. Nodes at the next, lower level, named Analyses, enable classification under more specific themes: in Figure 4, the Analysis Tree is opened under the Analysis **A498-series**, which contains results specific to the A498 cell line assays. The nodes at all levels below Projects and Analyses contain the actual results of analysis operations, and are arranged in a recursive, parent-child hierarchy of arbitrary depth. Thus in Figure 4, under **A498-series**, five generations of results are displayed. Note that each analysis result has a specific data type, indicated by the extension of its name and by a color-coded icon. A total of 33 data types are currently defined in Gecko.

### The analysis workflow for the A498 cell line data

**Figure 5:**
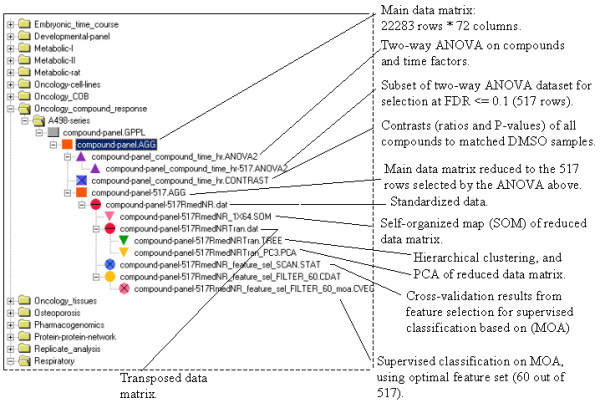
**Details of the analysis workflow for the A498 cell line data **(this is an expanded view of the Analysis Tree shown in Figure 4).

The analysis workflow of the A498 cell line data is indicated in an expanded view of the Analysis Tree (Figure 5). The analysis was started by creating a single data matrix out of the 72 independent scans which together constitute all data for the A498 series. The data matrix was created by a copy-and-paste submission of a spreadsheet containing the list of scans to the *Concatenate *tool, which then automatically assembled and normalized the relevant scan data. This operation resulted in two objects, a scan reference file, **compound_panel.GPPL **(grey square icon), containing the constitutive list of scans, and the data matrix itself, **compound_panel.AGG **(orange square icon). Note that these two objects were automatically inserted below the analysis node **A498-series**, with **compound_panel.AGG **inserted as a child of **compound_panel.GPPL**.

It is important to emphasize that the data matrix **compound-panel.AGG **is physically stored on the server platform. This centrality insures that all users have simultaneous access to ongoing analyses, and if desired, that they can collaborate in real-time, even when working from very different geographical locations (Figure 1).

The data matrix **compound_panel.AGG **has dimensions 22283 rows × 72 columns, with each row corresponding to a different Affymetrix qualifier on the HG_U133 chip (here the term "qualifier" is synonymous with Affymetrix "probe set"), and each column to a specific experimental sample. The associated *experimental design *[[4], p. 93] [[5], p. 219] of the A498 series, is also saved in the Gecko database in association with **compound_panel.AGG**, and is displayed in the Properties window (bottom window in Figure 4). The experimental design was originally specified in the spreadsheet submitted to the *Concatenate *tool, but can also be modified (or newly created) at any later time. It contains four factors, labeled *dose*, *time_hr, compound *and *moa*, corresponding to compound doseage, harvest time, compound name and compound mechanism of action, respectively.

**Figure 6:**
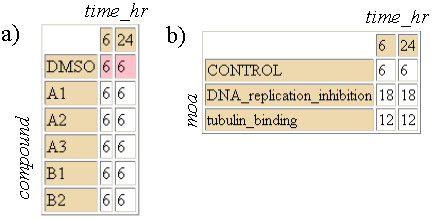
**The two-factorial designs for compound_panel.AGG**, resulting from the factor combinations a) (*compound *× *time_hr*) or b) (*moa *× *time_hr*). The number of replicates for every combination of levels is indicated in each cell of the tables. The pink cells in a) are flagged as reference cells for contrast calculations with all other cells in the corresponding column.

Based on a general experimental design, one can then automatically define in Gecko simpler two-factorial designs, by selection of the factors in the appropriate client interface. For instance, Figs. 6a and 6b display the two-factorial designs for **compound_panel.AGG **which result from the combinations (*compound *× *time_hr*) and (*moa *× *time_hr*), respectively. The number of replicates for every combination of levels is indicated in each cell of the tables. The factorial design (*compound *× *time_hr*) is of particular interest for finding genes with expression differentially regulated by the treatments with the different compounds, with or without concommittant time variation. In the A498 analysis workflow, this design was used to generate a two-way analysis of variance (ANOVA) [[5], p. 214] of **compound_panel.AGG**, resulting in the dataset **compound-panel_compound_time_hr.ANOVA2 **(Figure 5, purple triangle icon), which was again automatically inserted as a child of its parent dataset. The two-way ANOVA is conducted on a qualifier-by-qualifier basis, and results in a file contains 22283 rows, each row consisting of the P-values (and associated statistics) for the *compound, time_hr *and *compound *× *time_hr *effects for the corresponding qualifier.

Once created, **compound-panel_compound_time_hr.ANOVA2 **can be revisited for selection of statistically significant data using a generic utility called *Get Stats*. In particular, *Get Stats *internally computes receiver operating characteristics [[7], p. 48] for all the effects considered in the factorial design, and permits selection of significant qualifiers at a specified false-discovery rate (FDR)[8]. For instance, for a threshold FDR ≤ 0.05 used in conjunction with the *compound *effects, one finds that 517 qualifiers out of 22283 exhibit compound-related changes in expression. In the Analysis Tree, the data subset corresponding to these 517 qualifiers, **compound-panel_compound_time_hr-517.ANOVA2**, is automatically inserted as a child of the parent file. The operation parameters (effect used for selection and threshold FDR) which generated the subset are also saved, and are displayed in the Properties window for reference.

Following the ANOVA operations, the original data matrix, **compound_panel.AGG**, was then filtered to the rows corresponding to the 517 signifi-cant qualifiers contained in **compound-panel_compound_time_hr-517.ANOVA2**, in preparation for down-stream clustering and supervised classification operations. This step, implemented by the subsetting tool *Reduce on Qlist*, results in the filtered data matrix **compound_panel-517.AGG**.

Note that for all of the datasets discussed above, prior to each operation, a tentative output name was automatically created (typically by a concatenation of the input dataset name and of the name of the operation to be applied), and then presented to the user in a preview page. The tentative name can then be modified, if desired, before final submission.

### Clustering and supervised classification of the A498 cell line data

Several additional analysis steps were performed on the A498 series data, illustrating the use of complementary unsupervised (clustering) methods, as well as a supervised classification approach. Starting from the filtered data matrix **compound_panel-517.AGG **(Figure 5), and after row standardization [[11], p. 8] (**compound_panel-517RmedNR.dat**), three clustering methods were first applied, resulting in i) a self-organized map[9] of the data with 1 × 64 cluster geometry (**compound_panel-517_1 **× **64.SOM**), ii) a hierarchical clustering using average linkage [[10], p. 318] (**compound_panel-517.TREE**), and iii), a principal component analysis (PCA) [[11], p. 23] (**compound_panel-517.PCA**).

Supervised classification of the samples was also performed, using the gene expression *k*-nearest-neighbor classifier[12] integrated into Gecko. The classification was done on the basis of mechanism of action of the compounds (excluding controls, and regrouping 6 hour and 24 hour samples), resulting in a two-class problem with class labels "DNA replication inhibition" and "tubulin binding". The *Feature Selection *tool was first used, to compute the misclassification error as a function of the number of features (qualifiers) retained in the dataset, using the Fisher interclass separation [[13], p. 135] as a feature selection criterion and with misclassification error computed using "leave-one-group-out" (LOGO) cross-validation [[13], p. 219]. In each step of the LOGO procedure, all instances corresponding to a given compound are simultaneously removed and cross-classified by the remaining instances in the training set. Applied to each compound in turn, this resulted in 5 separate cross-classifications, each applied to the 12 held-out samples, with a tally of all misclassifications errors applied at the very end. The results were saved in **compound_panel-517RmedNR_feature_sel_SCAN.STAT**. An explicit *k*-nearest-neighbor classification, using an optimal set of 60 qualifiers determined by the feature selection step was then performed. The final classification results, including an internally generated PCA representation of the data, were automatically saved in the data set **compound_panel-517RmedNR_feature_sel_FILTER_60_moa.CVEC **(pink circle icon with × pattern).

### Visualization of analysis results

**Figure 7:**
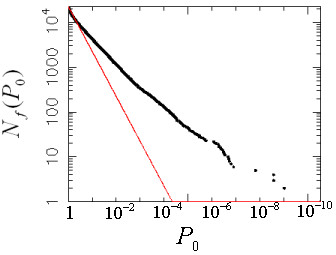
**Receiver operating characteristic for the selection of qualifiers according to *compound *effects **in the two-way ANOVA data set **compound-panel_compound_time_hr.ANOVA2**, with plot generated in the Gecko Browser window. The number *N*_*f *_(*P*_0_) of qualifiers found at a P-value less than or equal to *P*_0 _is plotted against *P*_0 _(note the log/-log scale used in the plot). The red line shows the trend expected under the null hypothesis of no compound effects (credits: pgplot graphics package).

**Figure 8:**
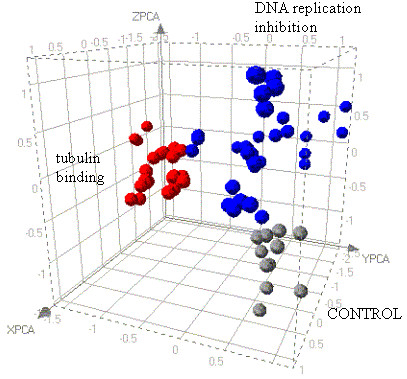
**Spotfire scatter plot for the three-dimensional principal component analysis of the training set **used for supervised classification, in the data set **compound_panel-517RmedNR_feature_sel_FILTER_60_moa.CVEC**, generated after data streaming to Spotfire. Samples are color coded in accordance to mechanism of action of the compound treatment, as indicated in the plot.

Gecko provides for flexible visualization of analysis results, with results either directly displayed in the client Browser window, or streamed to external visualization tools such as Spotfire[2]. In Figure 7, the receiver operating characteristic for the distribution of P-values according to *compound *effects in the two-way ANOVA (**compound-panel_compound_time_hr.ANOVA2**) is displayed in the Browser window. In Figure 8, after streaming to Spotfire, a PCA representation of data for the supervised classification **compound_panel-517RmedNR_feature_sel_FILTER_60_moa.CVEC **is displayed as a three-dimensional scatter plot.

## Conclusions

Constructed around the three organizing concepts of the Analysis Tree, the agglomeration syntax, and the pervasive use of experimental designs, Gecko has proven to be a robust analysis platform for a large and distributed scientific community. Gecko has allowed for flexible incorporation of new analysis methods over time, and has insured intelligible access to older, complex analyses, successfully answering the question of "where is my data?".

It should be emphasized that the analysis framework afforded by Gecko is general and not limited to gene expression data. Data can be uploaded from many other sources, and the analysis methods relevant to the new data types can also be incorporated as needed. Thus, methodologies for the analysis of protein-protein interaction data[22], or for the analysis of Gene Ontology, categorical data[23] have been integrated into Gecko in the past. It is now hoped that with its public release, many other uses will be found for this general analysis platform.

## Availability and requirements

All components of the Gecko software, including source code, are being made available as a package under SourceForge.net[1]. The Gecko project's home page will provide information regarding release schedules and availability. Interested parties may also directly contact the corresponding author (JT) for information.

Installation of the complete platform will require manual intervention as well as execution of the automated builds provided in the package. Manual intervention is required for installation of the required external software libraries (Perl modules, GNU software, graphics software, etc) as well as for setting up the run-time Gecko infrastructure (web server, servlet engine, Oracle data base). The automated builds provide for compilation of the C++ and Java source code, for creation of required flat-file directories, and for the creation of the database tables.

Existing installations of the Gecko platform are on Sun Enterprise UNIX servers running SunOS 2.8. Transposition to other operating systems, such as Linux, will thus require some additional "tuning" of components during installation. It should also be noted that the Gecko numerical analysis programs can be used in a standalone fashion (i.e. by execution from the command line), without requiring a complete installation of the platform.

## Authors' contributions

JT designed and implemented the early browser-based version of Gecko; he then focused on algorithm and tool development and implementation, alongside giving overall scientific direction for the design of the production version. AU designed and implemented the database. AM designed and built the Java servlets which process user requests, as well as implement many analysis tools and utilities. JC designed and built the client application. DX designed and implemented several statistical analysis algorithms. RN and MH have been involved in running, packaging and in creating automated builds of the Gecko platform. CB had a central role in promoting the Gecko platform and in scientific design input. SB initiated and oversaw development of the production version of Gecko, and was closely involved with the design of the earlier browser-based versions.
